# Template Method for Synthesizing Hierarchically Porous MIL-101(Cr) for Efficient Removal of Large Molecular Dye

**DOI:** 10.3390/ma15165763

**Published:** 2022-08-20

**Authors:** Minmin Zou, Hexin Zhu, Ming Dong, Tian Zhao

**Affiliations:** School of Packaging and Materials Engineering, Hunan University of Technology, Zhuzhou 412007, China

**Keywords:** MIL-101(Cr), SiO_2_, dye adsorption, template

## Abstract

As one of the most important prototypical chromium-based MOFs, MIL-101(Cr) is well-studied and widely employed in various scientific fields. However, due to its small capture window sizes and curved internal apertures, its application in large molecular removal is quite limited, and given its high stability and high synthetic temperature (>200 °C), it is difficult to achieve hierarchically porous MIL-101(Cr). In our study, hierarchically porous MIL-101(Cr) involving a high macro-/meso-/micropores ratio was designed and synthesized using acetic acid as an additive and silicon dioxide (SiO_2_) nanoparticles as a template. The optimal hierarchically porous MIL-101(Cr) (A-4) possessed a high specific surface area (2693 m^2^ g^−1^) and an abundant macro-/mesoporous structure with the addition of SiO_2_ of 200 mg. Compared with the control sample (A-0) with a less macro-/mesoporous structure, A-4 showed good adsorption properties for both coomassie brilliant blue R-250 (CBB, 82.1 mg g^−1^) and methylene blue (MB, 34.3 mg g^−1^) dyes, which were 1.36 times and 9.37 times higher than those of A-0. Moreover, A-4 also had good recyclability, and the removal rate of CBB was still higher than 85% after five cycles of adsorption.

## 1. Introduction

Different synthesis methods greatly influence the morphology, specific surface, and crystallinity of metal–organic framework materials (MOFs) [1]. The common synthesis methods of MOFs include hydrothermal synthesis [2,3], the solvent heat method [4,5], the microwave-assisted method [6], and the template method [7]. The template method is an important strategy for controlling the crystals’ morphology and size. It is divided into hard and soft templates, depending on the characteristics of the template itself and its domain-limiting ability [8,9]. In general, the template method is commonly used to prepare hierarchically porous MOFs (HP-MOFs), which improve the performance of MOF materials and expand their application fields [7,10]. The prefabricated template is directly added to the precursor solution of the MOFs material, and the MOFs crystals are then grown directly on the surface of the template [11]. The template occupies space in the crystal framework of MOFs, and the removal of the template results in the HP-MOFs containing extra porous structures such as mesopores or macropores [12,13].

The hierarchically porous materials are comprised of a multimodal hierarchically porous structure made of interconnected pores with different lengths, ranging from micro- (<2 nm) to meso- (2–50 nm) to macropores (>50 nm). Compared with conventional MOFs and microporous structures, HP-MOFs have a specific morphology and size, larger specific surface area, and greater pore volume [14,15]. HP-MOFs not only maintain the basic properties of the related pristine MOFs but also construct macro/mesoporous structures on the basis of micropores, which reduce macromolecular diffusion barriers and optimize the deficiencies of microporous pristine MOFs [16,17]. The HP-MOFs demonstrate excellent performance in the utilization of gas storage and separation [18], catalysis [19], drug mitigation [20], and adsorption [21,22,23]. Thus, the design and synthesis of HP-MOF materials with unique morphology and hierarchical pores are also crucial for many applications in specific fields (e.g., drug transport, adsorption, catalysis, etc.).

MIL-101(Cr) is a classical chromium-based MOF with an ultra-high specific surface area (SBET > 4000 m^2^ g^−1^) and excellent chemical/water stability. It is widely used in many fields, including catalysis [24,25], contaminant adsorption [26,27,28,29,30], detection [31,32], and drug transport [33,34]. MIL-101(Cr) is a typical mesoporous MOF with a maximum pore size of 34 Å. However, its biggest capture window is only ~16 Å, which is not conducive to the rapid diffusion and transport of molecules, especially in terms of applications for the transportation of large molecules. This greatly hinders the practical application of MIL-101(Cr) [35,36]. Hence, the synthesis of MIL-101(Cr) with a hierarchical pore structure using the template method not only preserves the excellent original properties of MIL-101(Cr) but also relieves the restriction on the applications involving large molecules.

With the development of industry and cities, environmental pollution is a growing concern. Specifically, the incomplete treatment of wastewater from the textile, leather, paper, printing, dye, plastic, and pharmaceutical industries can lead to serious pollution and harmful for human bodies [37,38,39,40]. Discharging organic dyes into the environment poses a significant risk to the environment and to human health due to the dyes’ toxicity and potential carcinogenicity. Methylene blue (MB) and coomassie brilliant blue R-250 (CBB) are the most commonly used dyes in the textile industry, and they are toxic and hard-to-degrade organic pollutants [38,41]. Diverse approaches for the removal of dyes from wastewater have been developed, including adsorption, degradation, coagulation, and sedimentation [42,43,44]. The adsorption method is one of the most commonly used methods due to its high efficiency, economic feasibility, and simplicity of equipment [45]. Due to its high porosity and stability, MIL-101(Cr) is widely used as an adsorbent for the adsorption/removal of dyes from water. For instance, Zhang [46] reported that the charge and size of MIL-101 (Cr) greatly affect its sorption performance. In their study, the positively charged MIL-101(Cr) hardly adsorbed the cationic dye methylene blue (MB), and the adsorption of the more negatively charged and aromatic ringed anionic dye congo red (CR) (1367.1 mg g^−1^) was also significantly higher than that of methyl orange (MO) (455.2 mg g^−1^). Mahmoodi [47] found that MIL-101 (Cr) exhibited good adsorption capacity for two dyes, direct red 80 (DR80) and acid blue 92 (AB92), in an aqueous solution, with maximum adsorption capacities of 227 and 185 mg g^−1^, respectively. Shen et al. [48] reported the adsorption removal of methyl orange and methylene blue from aqueous solutions using hierarchical pore MIL-101(Cr) synthesized with different mineralizing agents. The adsorption of MB by MIL-101(Cr) synthesized using sodium acetate was much higher than that of MIL-101(Cr) synthesized using HF, roughly 3.2 times higher. For the removal of MB, MIL-101(Cr) containing a mesoporous structure has a higher removal of dyes than that of conventional microporous MIL-101(Cr), and the higher the mesoporous ratio is, the better the adsorption performance of MIL-101(Cr) is.

In our previous study, a template-free method was reported for producing nanofused hierarchically porous MIL-101(Cr) (A-0) with the presence of acetic acid [25]. However, due to MIL-101(Cr)’s small pores and low meso-/micropores ratio, its capability to remove large dye molecules, such as CBB or MB, is limited [25]. Herein, we propose a template method for the design and synthesis of HP-MIL-101(Cr) with a high meso-/micro-pores ratio, which shows a high adsorption capacity toward large toxic dyes. Compared with the HP-MIL-101(Cr) and template-free method (A-0), the obtained HP-MIL-101(Cr) with template method (A-4) exhibited a high adsorption capacity for the large dyes of CBB and MB, specifically, 1.36 times and 9.37 times higher than that of A-0, respectively.

## 2. Experimental Section

### 2.1. Raw Materials and Reagents

Cr(NO_3_)_3_·9H_2_O (99.5%, AR), terephthalic acid (H_2_BDC, 99%, AR), N, N-dimethylformamide (DMF, 99.5%, AR), acetic acid (99.5%, AR), nano-silica (SiO_2_, 99.8%, 15 nm), methylene blue (MB, AR), Coomassie Brilliant Blue R-250 (CBB, AR), and anhydrous ethanol were all purchased from Aladdin Chemical Reagent Co. All chemicals were used as obtained from commercial sources without further purification.

### 2.2. Characterization and Analysis

X-ray diffraction tests were performed on an X-ray diffractometer (D8 Advance, Bruker, Karlsruhe, Germany). A scanning electron microscope (Gemini 300, Zeiss, Jena, Germany) and transmission electron microscope (Talos F200X, FEI, Hillsboro, OR, USA) were used to characterize the samples’ microscopic morphology and particle size. The BET-specific surface area and pore volume of the samples were determined via nitrogen adsorption/desorption experiments with a fully automated specific surface area and porosity analyzer (NOVA-4200e, Quantachrome, Boynton Beach, FL, USA). A UV-Vis spectrophotometer (UV-2600, Shimadzu, Suzhou, China) was employed to monitor the concentration of dyes during the adsorption experiments. All samples followed the same pretreatment before measurements.

### 2.3. Synthesis and Purification of Samples

To synthesize MIL-101 (Cr), we added 800 mg of Cr(NO_3_)_3_·9H_2_O (2 mmol), 332 mg of H_2_BDC (2 mmol), 8 mmol of acetic acid, and 0~200 mg SiO_2_ (15 nm) to 10 ml of deionized water and stirred for 30 min. The mixture was then transferred into a polytetrafluoroethylene (PTFE) autoclave (20 mL, Youmai, Shanghai, China) and placed in the oven at 200 °C for 8 h. After the reaction, the autoclave was slowly cooled down to ambient temperature, and the green product was collected for purification. Samples of 0 mg, 50 mg, 100 mg, 150 mg, 200 mg, and 250 mg of SiO_2_ were named A-0, A-1, A-2, A-3, A-4, and A-5, respectively. However, adding 250 mg of SiO_2_ into the autoclave would have filled the whole container and overflowed when stirred. Thus, for security consideration, an A-5 experiment was not conducted, and 200 mg of SiO_2_ was the largest sample in our study.

For purification, the green products were gathered and washed with hot DMF (70 °C) and hot ethanol (60 °C) twice (1 h each). The samples were the washed with HF aqueous (1%) for 12 h to remove SiO_2_. Finally, the solids were rinsed with deionized water at least 5 times to remove residual HF. The final products were dried in a vacuum oven at 120 °C for 12 h.

### 2.4. Material Adsorption Performance Test

#### 2.4.1. Dye Adsorption Experiments

We added 2.5 mg of adsorbent to 10 mL of dyes (MB and CBB) with different initial concentrations at ambient temperature. The mixture was then placed into an ultrasonicator and shaken for an appropriate time (~10 min). After centrifugation, the supernatant was taken for an absorbance test using a UV spectrophotometer. The remaining concentration of dyes in the supernatant was measured using a UV spectrophotometer.

#### 2.4.2. Adsorption Amount and Adsorption Model

1. Adsorption capacity

The adsorption capacity *q_e_* (mg g^−1^) of the adsorbent for the dyes was calculated using the formula shown in Equation (1) [49].

(1) $$q_{e}=\frac{v\left(c_{0}-c_{e}\right)}{m}$$

where *c_0_* and *c_e_* are the initial and equilibrium concentrations of the dye’s aqueous solution (mg L^−1^), respectively. *v* is the volume of the dye solution (L), *m* is the mass of the adsorbent (g), and *q_e_* is the adsorption amount of the adsorbent on the dye molecules at adsorption equilibrium (mg g^−1^).

2. Kinetic study of adsorption
  (1) Pseudo-first-order kinetic model

The pseudo-first-order kinetic model considers a physical adsorption process between the adsorbent and the adsorbate [50,51]. It assumes that a diffusion step controls the arrival of the adsorbate from the solution to the adsorbent surface and that there is only one active site on the adsorbent surface. The linear expressions of the pseudo-first-order kinetic mode model are shown in Equation (2) [52].

(2) $$\ln(q_{e}-q_{t})=\ln q_{e}-k_{1}t$$

where *t* is the adsorption time (min), *q_e_* is the adsorbed amount of adsorbent on the adsorbent’s surface (mg g^−1^), *q_t_* is the adsorption amount of the dye molecule by the adsorbent at moment *t* (mg g^−1^), and *k_1_* (min^−1^) is the adsorption rate constant for the pseudo-first-order kinetic model.

  (2) Pseudo-second-order kinetic model

The pseudo-second-order kinetic model considers the chemisorption process between the adsorbent and the adsorbate [53,54]. It assumes that the chemisorption mechanism controls the adsorption rate and that there are two binding sites on the adsorbent surface. The linear expressions of the pseudo-second-order kinetic model are shown in Equation (3) [53].

(3) $$\frac{t}{q_{t}}=\frac{1}{k_{2}q_{e}^{2}}+\frac{t}{q_{e}}$$

where *t* is the adsorption time (min), *q_e_* is the adsorbed amount of the adsorbent on the adsorbent’s surface (mg g^−1^), *q_t_* is the adsorption amount of the dye molecule by the adsorbent at moment *t* (mg g^−1^), and *k_2_* (g mg^−1^ min^−1^) is the adsorption rate constant for the pseudo-second-order kinetic model.

3. Isothermal adsorption model
  (1) Langmuir model

The Langmuir model is one of the commonly used adsorption models. It assumes that adsorption occurs uniformly on all active sites of the adsorbent and that the adsorbate is adsorbed on the surface of the adsorbent as a single molecular layer [55]. The linear expression of the Langmuir model is shown in Equation (4) [56].

(4) $$\frac{c_{e}}{q_{e}}=\frac{c_{e}}{q_{m}}+\frac{1}{k_{L}q_{m}}$$

where *q_m_* is the maximum adsorption of the adsorbent on the adsorbent’s surface (mg g^−1^), *c_e_* is the concentration of the solution at the adsorbent’s surface when it reaches adsorption equilibrium (mg L^−1^), and *k_L_* is the Langmuir equilibrium constant (L mg^−1^).

  (2) Freundlich model

The Freundlich model is based on the facts that the surface energy of the adsorbent is inhomogeneous and that the adsorption of the adsorbent on the adsorbent’s surface is not a single monomolecular layer adsorption, but a multimolecular layer adsorption [57]. The linear expression of the Freundlich model is shown in (5) as follows [58].

(5) $$\ln q_{e}=\ln k_{F}+\frac{1}{n}\ln c_{e}$$

where *q_e_* is the adsorbed amount of adsorbent on the adsorbent’s surface (mg g^−1^), *c_e_* is the concentration of the solution at the adsorption equilibrium on the adsorbent’s surface (mg L^−1^), *k_F_* is the Freundlich equilibrium constant representing the adsorption capacity (mg g^−1^), and *n* is the Freundlich equilibrium constant for the adsorption strength.

## 3. Results

**Figure 1:**
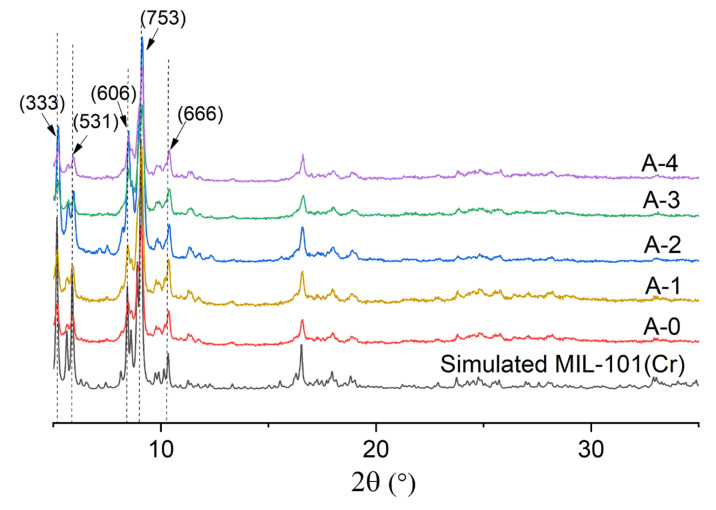
XRD patterns of the samples compared with the simulated XRD pattern.

X-ray diffraction (XRD) plots showed that all of the experimentally prepared samples were highly consistent with the simulated XRD pattern generated from the deposited X-ray data file at the Cambridge Structure Database (CSD-Refcode OCUNAK) using the Mercury program. Thus, all the samples were confirmed to be pure MIL-101(Cr) [59] (Figure 1).

**Figure 2:**
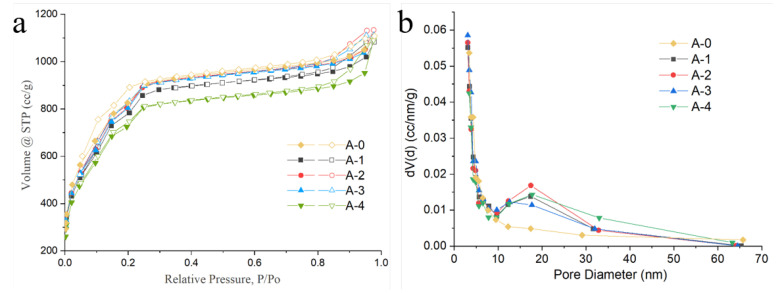
(**a**) Nitrogen adsorption and desorption curves of samples. (**b**) BJH pore size distribution curves of each sample.

The nitrogen adsorption–desorption curves of the samples are shown in Figure 2, and the adsorption and desorption curves of the samples did not completely overlap. The hysteresis loops indicated that the pore structures of the samples were irregular and contained two or more types of pore structures [60]. The pore structure information of each sample is shown in Table 1. The specific surface area of the MIL-101(Cr), synthesized using only acetic acid as the modulating agent, was 3006 m^2^ g^−1^. However, the addition of SiO_2_ decreased the specific surface area of MIL-101(Cr). In particular, when the addition amount of SiO_2_ was 200 mg, the BET-specific surface area of A-4 was 2693 m^2^ g^−1^. Interestingly, adjusting the amount of SiO_2_ increased the ratio of macro-mesopores to micropores (S_macro-meso_/S_micro_). The S_macro-meso_/S_micro_ ratio increased from 4.62:1 to 12.8:1 when the addition amount of SiO_2_ was raised from 50 mg to 200 mg (see Table 1). Combined with the Barret–Joyner–Halenda (BJH) pore size distribution curves of the samples (Figure 2b), we found that involving the addition of SiO_2_ as a template created more macro-/mesopores. Thus, A-4 had the highest S_macro-meso_/S_micro_ ratio among all samples. The specific surface area was influenced by many factors, such as particle size and pore structure. In general, the smaller the particle size was and the simpler the pore structure of MIL-101(Cr) was, the higher its specific surface area was. In our study, A-4 contained the most abundant macro-/mesoporous structure and the most relatively large particle size; thus, it possessed relatively low specific surface area.

**Figure 3:**
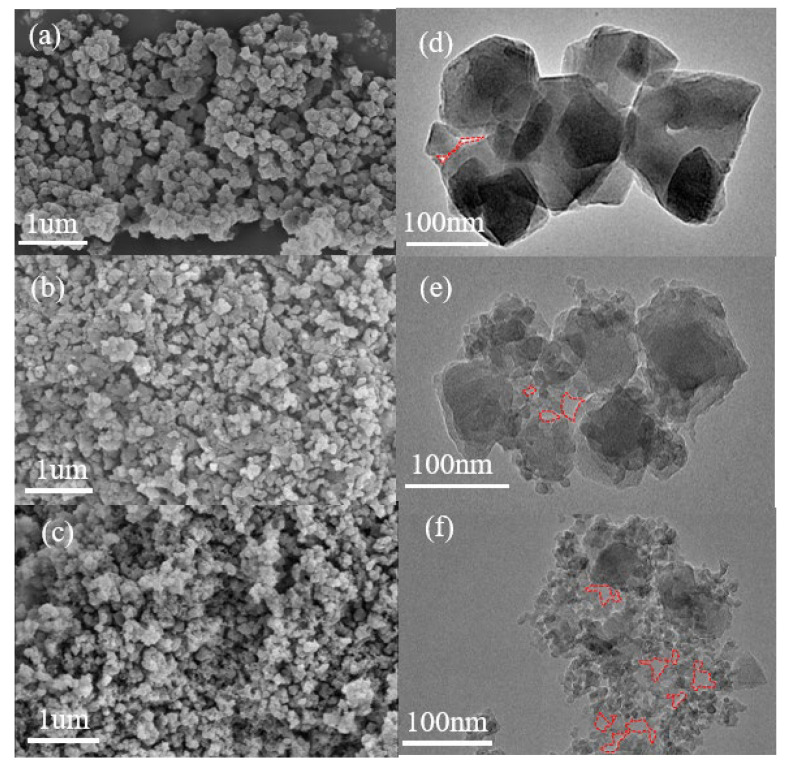
(**a**,**d**) SEM and TEM images of sample A-0. (**b**,**e**) SEM and TEM images of sample A-2. (**c**,**f**) SEM and TEM images of sample A-4. The red dashed area in the TEM image represents the mesoporous structure formed by crystal nanofusion.

Scanning electron microscope (SEM) and transmission electron microscope (TEM) images of the samples showed that the SiO_2_-free sample A-0 presented a uniform particle size of ~138 nm with a relatively regular morphology (Figure 3a,d and Figure S1a in the Supplementary Section). There was a weak nanofusion phenomenon at the boundary of the crystals and a trace of mesoporous structure (red dashed area). Samples A-2 and A-4 showed irregular polyhedral morphology, with the adjacent crystals showing nanofusion and forming mesoporous structures (Figure 3b,c,e,f). The difference was that A-4 had a higher degree of nanofusion and formed more abundant mesoporous structures (red dashed area).

**Figure 4:**
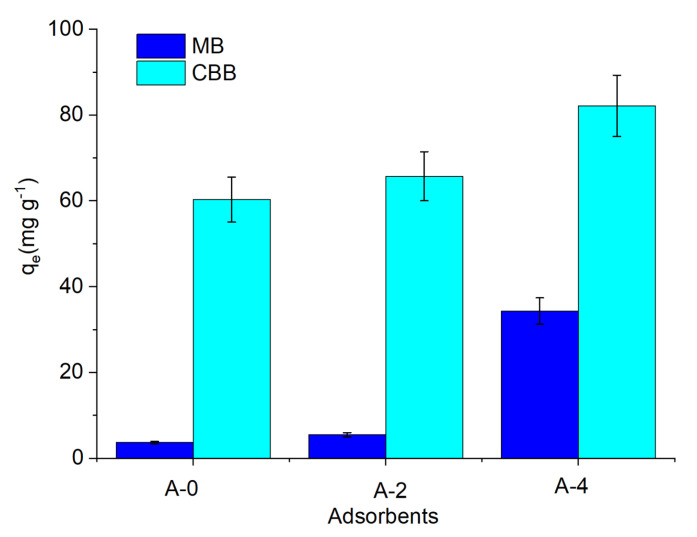
Adsorption of CBB and MB by A-0, A-2, and A-4 (test conditions: concentration of dye solution 50 mg L^−1^, adsorption time 12 h, adsorbent mass 2.5 mg).

The adsorption properties of A-0, A-2, and A-4 were tested using aqueous solutions of methylene blue (7.9 Å × 16.3 Å, Figure S2a) and Coomassie Brilliant Blue (15.1 Å × 24.1 Å, Figure S2b in the Supplementary Section). A-4 showed the best adsorption capability toward CBB and MB among all of the samples, with the adsorption amounts of 82.1 mg g^−1^ and 34.3 mg g^−1^ (Figure 4), respectively, attributable to its high S_macro-meso_/S_micro_ ratio structure. The more mesopores that a sample had, the better that the large molecular dyes were adsorbed. Hence, we were not surprised that A-4, which possessed the highest S_macro-meso_/S_micro_ ratio, exhibited the best adsorption capacity for the large molecular dyes in CBB and MB.

**Figure 5:**
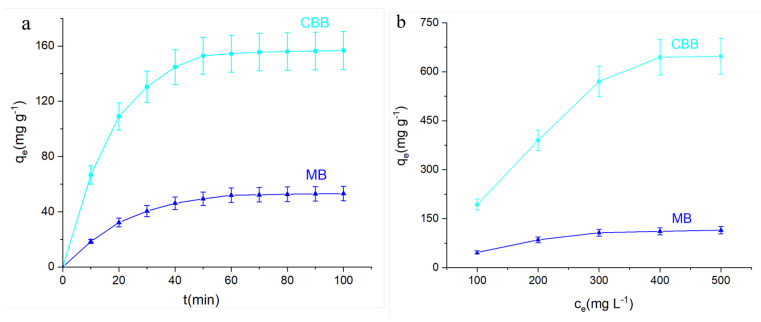
(**a**) Time adsorption curves of A-4 on CBB and MB (adsorbent 5 mg, dye concentration 100 mg L^−1^, room temperature). (**b**) The maximum adsorption capacity of A-4 for CBB and MB at different initial concentrations. The adsorption time was over 24 h.

The effects of time and dye concentration on the adsorption capacity of A-4 were investigated, and the results are presented in Figure 5. During the first 60 min was the rapid adsorption phase, when the adsorption amount increased rapidly from 0 mg g^−1^ to 154.5 mg g^−1^ for CBB and from 0 mg g^−1^ to 50.0 mg g^−1^ for MB. This was followed by a slow increase until it finally reached the adsorption equilibrium, after which the adsorption amount was essentially unchanged. This was considered to be the maximum adsorption. Figure 5b shows the adsorption capacity of A-4 for different initial concentrations of CBB and MB (100 mg L^−1^~500 mg L^−1^ with an adsorption time of 24 h). As the dye concentration increased, the adsorption of A-4 on the dye molecules also increased, while the adsorption increment decreased. It is noteworthy that the adsorption capability of A-4 for CBB is much higher than that of MB (Figure 5). This phenomenon may be caused by the positive charge of A-4 (Figure S3 in the Supplementary Section) and the negative charge of the anionic dye CBB, which induces a strong electrostatic interaction between them. In contrast, since there was no such electrostatic interaction between the cationic dye MB and A-4, A-4 had better adsorption of CBB.

**Figure 6:**
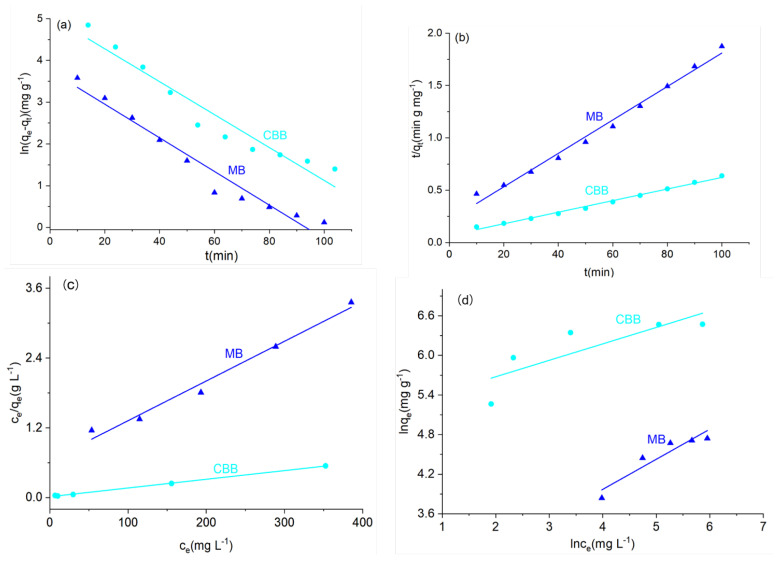
(**a**) Pseudo-first-order kinetic data. (**b**) Pseudo-second-order kinetic data. The dyes’ adsorption of A-4 analyzed using the (**c**) Langmuir model and the (**d**) Freundlich model.

The results of fitting the above adsorption data using adsorption kinetics and adsorption isotherms are shown in Figure 6 and Table S1 in the Supplementary Section. The correlation coefficients R^2^ of the pseudo-second-order kinetic fits of A-4 for the adsorption processes of CBB and MB were 0.993 and 0.989, which were higher than those of the pseudo-first-order kinetic fits (0.936 and 0.956). Furthermore, the theoretical adsorption amount of the pseudo-second-order kinetic was close to the actual adsorption amount, indicating that the adsorption processes of A-4 on CBB and MB were more consistent with the secondary kinetic model. For the adsorption isotherms, the correlation coefficients R^2^ of the Langmuir model were 0.999 (CBB) and 0.982 (MB), respectively, much higher than those of the Freundlich model (0.689 and 0.888); the theoretical values were also very close to the actual values. This indicated that the Langmuir model could more accurately describe the adsorption process of A-4 on CBB and MB. Additionally, the cyclicity of A-4 was conducted using CBB as the model, and the results are presented in Figure S4 in the Supplementary Section [21,48,61,62,63,64,65]. After five cycles, the removal efficiency of A-4 for CBB still reached more than 85%, indicating that A-4 had good reusability.

## 4. Conclusions

In summary, hierarchically porous MIL-101(Cr) containing a rich macro-/mesoporous structure can be obtained by using SiO_2_ as a template, whose S_macro-__meso_/S_micro_ ratio could be up to 12.8:1. Under optimal synthesis conditions, the obtained HP-MIL-101(Cr) (A-4) possessed a relatively high specific surface area and a large molecular dyes’ adsorption capacity toward CBB and MB. Compared with non-template synthesized MIL-101(Cr) (A-0), the significantly enhanced adsorption capability of A-4 for dyes was attributed to the richer macro-/mesoporous structure, which could notably increase the mass transfer capacity of MIL-101(Cr) in aqueous solutions. The demonstrated template synthesis broadened the application of MIL-101(Cr) in effluent treatment fields, especially in terms of large molecular organic dyes’ removal.

## Figures and Tables

**Table 1:** The pore structure information of each sample.

Sample	*S*_BET_ (m^2^ g^−1^) ^a^	*S*_Langmuir_ (m^2^ g^−1^)	*V*_pore_ (cm^3^·g^−1^) ^b^	S_macro-__meso_/S_micro_^c^
A-0	3006	4269	1.71	4.62:1
A-1	2873	4079	1.67	6.17:1
A-2	2989	4243	1.75	7.35:1
A-3	2979	4254	1.72	6.80:1
A-4	2693	3668	1.69	12.8:1

^a^ Calculated from the N_2_ adsorption/desorption curves in the relative pressure range of 0.05 < p/p_0_ < 0.2, with an error of ± 50 m^2^ g^−1^. ^b^ Calculated from the N_2_ adsorption/desorption curves at 77 K and a relative pressure of p/p_0_ = 0.95. ^c^ Calculated from the BJH desorption curves by integrating over the micropore and mesopore intervals S_macro-meso_/S_micro_.

## Data Availability

Not applicable.

## Supplementary Materials

The following supporting information can be downloaded at: https://www.mdpi.com/article/10.3390/ma15165763/s1, Figure S1: Particle size distribution of the samples: (a) A-0, (b) A-2, (c) A-4; Figure S2: Molecular diagrams of (a) methylene blue dye and (b) coomassie brilliant blue dye; Figure S3: Zeta potential of A-4; Table S1: Fitting parameters of Langmuir, Freundlich, and Temkin models for sample A-4; Figure S4: Cycle adsorption efficiency of A-4 adsorbed CBB.

## References

[1] Bhattacharjee S., Chen C., Ahn W.S. (2014). Chromium terephthalate metal–organic framework MIL-101: Synthesis, functionalization, and applications for adsorption and catalysis. RSC Adv..

[2] Zhao T., Yang L., Feng P., Gruber I., Janiak C., Liu Y. (2018). Facile synthesis of nano-sized MIL-101(Cr) with the addition of acetic acid. Inorg. Chim. Acta.

[3] Zhao T., Zhu H., Dong M., Tang S., Luo M., Li X. (2022). Low-temperature and additive-free synthesis of spherical MIL-101(Cr) with enhanced dye adsorption performance. Inorganics.

[4] Zhang Z., Wang H., Chen X., Zhu C., Wei W., Sun Y. (2015). Chromium-based metal–organic framework/mesoporous carbon composite: Synthesis, characterization and CO_2_ adsorption. Adsorption.

[5] Fallah M., Sohrabnezhad S. (2019). Study of synthesis of mordenite zeolite/MIL-101 (Cr) metal–organic framework compounds with various methods as bi-functional adsorbent. Adv. Powder Technol..

[6] Soltanolkottabi F., Talaie M.R., Aghamiri S., Tangestaninejad S. (2019). Introducing a dual-step procedure comprising microwave and electrical heating stages for the morphology-controlled synthesis of chromium-benzene dicarboxylate, MIL-101(Cr), applicable for CO_2_ adsorption. J. Environ. Manag..

[7] Yang L.T., Qiu L.G., Hu S.M., Jiang X., Xie A.J., Shen Y.H. (2013). Rapid hydrothermal synthesis of MIL-101(Cr) metal–organic framework nanocrystals using expanded graphite as a structure-directing template. Inorg. Chem. Commun..

[8] Xie Y., Kocaefe D., Chen C., Kocaefe Y. (2016). Review of research on template methods in preparation of nanomaterials. J. Nanomater..

[9] Zhang W., Cheng R.R., Bi H.H., Lu Y.H., Ma L.B., He X.J. (2021). A review of porous carbons produced by template methods for supercapacitor applications. New Carbon Mater..

[10] Huang X.X., Qiu L.G., Zhang W., Yuan Y.P., Jiang X., Xie A.J., Shen Y.H., Zhu J.F. (2012). Hierarchically mesostructured MIL-101 metal–organic frameworks: Supramolecular template-directed synthesis and accelerated adsorption kinetics for dye removal. CrystEngComm.

[11] Xi J., Li H., Xi J., Tan S., Zheng J., Tan Z. (2020). Preparation of high porosity biochar materials by template method: A review. Environ. Sci. Pollut. Res. Int..

[12] Wu Y.N., Li F., Zhu W., Cui J., Tao C.A., Lin C., Hannam P.M., Li G. (2011). Metal-organic frameworks with a three-dimensional ordered macroporous structure: Dynamic photonic materials. Angew. Chem. Int. Ed. Engl..

[13] Chen S., Zhang L., Zhang Z., Qian G., Liu Z., Cui Q., Wang H. (2018). Study on the desorption process of n-heptane and methyl cyclohexane using UiO-66 with hierarchical pores. ACS Appl. Mater. Interfaces.

[14] Cui J., Gao N., Yin X., Zhang W., Liang Y., Tian L., Zhou K., Wang S., Li G. (2018). Microfluidic synthesis of uniform single-crystalline MOF microcubes with a hierarchical porous structure. Nanoscale.

[15] Laha S., Chakraborty A., Maji T.K. (2020). Synergistic role of microwave and perturbation toward synthesis of hierarchical porous MOFs with tunable porosity. Inorg. Chem..

[16] Zheng M., Tang H., Li L., Hu Q., Zhang L., Xue H., Pang H. (2018). Hierarchically nanostructured transition metal oxides for lithium-Ion batteries. Adv. Sci. (Weinh).

[17] Cai G., Yan P., Zhang L., Zhou H.C., Jiang H.L. (2021). Metal-organic framework-based hierarchically porous materials: Synthesis and applications. Chem. Rev..

[18] Shi Y., Huang J., Chen L., Wang S., Xu J., Zhu F., Cui S., Zheng J., Ouyang G. (2022). MOF-74/polystyrene-derived Ni-doped hierarchical porous carbon for structure-oriented extraction of polycyclic aromatic hydrocarbons and their metabolites from human biofluids. J. Hazard Mater..

[19] Duan C., Liang K., Lin J., Li J., Li L., Kang L., Yu Y., Xi H. (2021). Application of hierarchically porous metal-organic frameworks in heterogeneous catalysis: A review. Sci. China Mater..

[20] Abedi M., Abolmaali S.S., Heidari R., Mohammadi Samani S., Tamaddon A.M. (2021). Hierarchical mesoporous zinc-imidazole dicarboxylic acid MOFs: Surfactant-directed synthesis, pH-responsive degradation, and drug delivery. Int. J. Pharm..

[21] Zhao T., Zhu H., Geng W., Zou M., Dong M., Ying J. (2022). Morphology control synthesis of Cr-benzenedicarboxylate MOFs for the removal of methylene blue. J. Solid State Chem..

[22] Khan N.A., Hasan Z., Jhung S.H. (2013). Adsorptive removal of hazardous materials using metal-organic frameworks (MOFs): A review. J. Hazard Mater..

[23] Zhang Z., Chen Y., Hu C., Zuo C., Wang P., Chen W., Ao T. (2021). Efficient removal of tetracycline by a hierarchically porous ZIF-8 metal organic framework. Environ. Res..

[24] Tang Y., Yin X., Mu M., Jiang Y., Li X., Zhang H., Ouyang T. (2020). Anatase TiO_2_@MIL-101(Cr) nanocomposite for photocatalytic degradation of bisphenol A. Colloids Surf. A Physicochem. Eng. Asp..

[25] Zhao T., Li S., Xiao Y.X., Janiak C., Chang G., Tian G., Yang X.Y. (2020). Template-free synthesis to micro-meso-macroporous hierarchy in nanostructured MIL-101(Cr) with enhanced catalytic activity. Sci. China Mater..

[26] Zhang W., Zhang R.Z., Yin Y., Yang J.M. (2020). Superior selective adsorption of anionic organic dyes by MIL-101 analogs: Regulation of adsorption driving forces by free amino groups in pore channels. J. Mol. Liq..

[27] Liu L., Ge J., Yang L.T., Jiang X., Qiu L.G. (2016). Facile preparation of chitosan enwrapping Fe_3_O_4_ nanoparticles and MIL-101(Cr) magnetic composites for enhanced methyl orange adsorption. J. Porous Mater..

[28] Huang X., Hu Q., Gao L., Hao Q., Wang P., Qin D. (2018). Adsorption characteristics of metal–organic framework MIL-101(Cr) towards sulfamethoxazole and its persulfate oxidation regeneration. RSC Adv..

[29] Mirsoleimani-azizi S.M., Setoodeh P., Samimi F., Shadmehr J., Hamedi N., Rahimpour M.R. (2018). Diazinon removal from aqueous media by mesoporous MIL-101(Cr) in a continuous fixed-bed system. J. Environ. Chem. Eng..

[30] Joseph L., Saha M., Kim S., Jun B.M., Heo J., Park C.M., Jang M., Flora J.R.V., Yoon Y. (2021). Removal of Cu^2+^, Cd^2+^, and Pb^2+^ from aqueous solution by fabricated MIL-100(Fe) and MIL-101(Cr): Experimental and molecular modeling study. J. Environ. Chem. Eng..

[31] Zhao X., Wang Y., Li J., Huo L., Huang H., Bai J., Peng Y., Li S., Han D., Ren S. (2021). A fluorescence aptasensor for the sensitive detection of T-2 toxin based on FRET by adjusting the surface electric potentials of UCNPs and MIL-101. Anal. Chim. Acta.

[32] Haghighi E., Zeinali S. (2020). Formaldehyde detection using quartz crystal microbalance (QCM) nanosensor coated by nanoporous MIL-101(Cr) film. Microporous Mesoporous Mater..

[33] Wang Y., Jia M., Wu X., Wang T., Wang J., Hou X. (2019). PEG modified column MIL-101(Cr)/PVA cryogel as a sorbent in stir bar solid phase extraction for determination of non-steroidal anti-inflammatory drugs in water samples. Microchem. J..

[34] Gordon J., Kazemian H., Rohani S. (2015). MIL-53(Fe), MIL-101, and SBA-15 porous materials: Potential platforms for drug delivery. Mater. Sci. Eng. C Mater. Biol. Appl..

[35] Huang H., Li J.R., Wang K., Han T., Tong M., Li L., Xie Y., Yang Q., Liu D., Zhong C. (2015). An in situ self-assembly template strategy for the preparation of hierarchical-pore metal-organic frameworks. Nat. Commun..

[36] Xuan W., Zhu C., Liu Y., Cui Y. (2012). Mesoporous metal-organic framework materials. Chem. Soc. Rev..

[37] Tan K.B., Vakili M., Horri B.A., Poh P.E., Abdullah A.Z., Salamatinia B. (2015). Adsorption of dyes by nanomaterials: Recent developments and adsorption mechanisms. Sep. Purif. Technol..

[38] Liu Q., Yu H., Zeng F., Li X., Sun J., Li C., Lin H., Su Z. (2021). HKUST-1 modified ultrastability cellulose/chitosan composite aerogel for highly efficient removal of methylene blue. Carbohydr. Polym..

[39] Cassano A., Molinari R., Romano M., Drioli E. (2001). Treatment of aqueous effluents of the leather industry by membrane processes A review. J. Membr. Sci..

[40] Zou M., Dong M., Luo M., Zhu H., Zhao T. (2022). Nanofused hierarchically porous MIL-101(Cr) for enhanced methyl orange removal and improved catalytic activity. Materials.

[41] Sharma G., Naushad M., Kumar A., Rana S., Sharma S., Bhatnagar A.J., Stadler F., Ghfar A.A., Khan M.R. (2017). Efficient removal of coomassie brilliant blue R-250 dye using starch/poly(alginic acid-cl-acrylamide) nanohydrogel. Process Saf. Environ. Prot..

[42] Aragaw T.A., Bogale F.M. (2021). Biomass-based adsorbents for removal of dyes from wastewater: A review. Front. Environ. Sci..

[43] Cai Z., Sun Y., Liu W., Pan F., Sun P., Fu J. (2017). An overview of nanomaterials applied for removing dyes from wastewater. Environ. Sci. Pollut. Res. Int..

[44] Mishra S., Cheng L., Maiti A. (2021). The utilization of agro-biomass/byproducts for effective bio-removal of dyes from dyeing wastewater: A comprehensive review. J. Environ. Chem. Eng..

[45] Song C., Li R., Fan Z., Liu Q., Zhang B., Kitamura Y. (2020). CO_2_/N_2_ separation performance of Pebax/MIL-101 and Pebax /NH2-MIL-101 mixed matrix membranes and intensification via sub-ambient operation. Sep. Purif. Technol..

[46] Zhang W., Zhang R.Z., Huang Y.Q., Yang J.M. (2018). Effect of the synergetic interplay between the electrostatic interactions, size of the dye molecules, and adsorption sites of MIL-101(Cr) on the adsorption of organic dyes from aqueous solutions. Cryst. Growth Des..

[47] Mahmoodi N.M., Taghizadeh M., Taghizadeh A. (2018). Ultrasound-assisted green synthesis and application of recyclable nanoporous chromium-based metal-organic framework. Korean J Chem Eng.

[48] Shen T., Luo J., Zhang S., Luo X. (2015). Hierarchically mesostructured MIL-101 metal–organic frameworks with different mineralizing agents for adsorptive removal of methyl orange and methylene blue from aqueous solution. J. Environ. Chem. Eng..

[49] Li J., Wang X., Zhao G., Chen C., Chai Z., Alsaedi A., Hayat T., Wang X. (2018). Metal-organic framework-based materials: Superior adsorbents for the capture of toxic and radioactive metal ions. Chem. Soc. Rev..

[50] Wang H., Lustig W.P., Li J. (2018). Sensing and capture of toxic and hazardous gases and vapors by metal-organic frameworks. Chem. Soc. Rev..

[51] Xu J., Cao Z., Zhang Y., Yuan Z., Lou Z., Xu X., Wang X. (2018). A review of functionalized carbon nanotubes and graphene for heavy metal adsorption from water: Preparation, application, and mechanism. Chemosphere.

[52] Wu X., Zhang Z., Xia C., Chen B., Jin X., Huang Z., Liu Y.g., Fang M., Min X. (2017). Magnetically recoverable Ni@C composites: The synthesis by carbonization and adsorption for Fe^3+^. J. Alloys Compd..

[53] Laciste M.T., de Luna M.D.G., Tolosa N.C., Lu M.C. (2020). Effect of calcination time of a quadruple-element doped titania nanoparticles in the photodegradation of gaseous formaldehyde under blue light irradiation. Chemosphere.

[54] Huang Y.D. (2018). Comments on “Magnetically recoverable Ni@C composites: The synthesis by carbonization and adsorption for Fe^3+^”. J. Alloys Compd..

[55] Aigbe U.O., Onyancha R.B., Ukhurebor K.E., Obodo K.O. (2020). Correction: Removal of fluoride ions using a polypyrrole magnetic nanocomposite influenced by a rotating magnetic field. RSC Adv..

[56] Tan S., Saito K., Hearn M.T.W. (2021). Isothermal modelling of protein adsorption to thermo-responsive polymer grafted Sepharose Fast Flow sorbents. J. Sep. Sci..

[57] Chen H., Zhao J. (2009). Adsorption study for removal of congo red anionic dye using organo-attapulgite. Adsorption.

[58] Ezzati R. (2020). Derivation of Pseudo-First-Order, Pseudo-Second-Order and Modified Pseudo-First-Order rate equations from Langmuir and Freundlich isotherms for adsorption. Chem. Eng. J..

[59] Zhao T., Jeremias F., Boldog I., Nguyen B., Henninger S.K., Janiak C. (2015). High-yield, fluoride-free and large-scale synthesis of MIL-101(Cr). Dalton Trans.

[60] Thommes M., Kaneko K., Neimark A.V., Olivier J.P., Rodriguez-Reinoso F., Rouquerol J., Sing K.S.W. (2015). Physisorption of gases, with special reference to the evaluation of surface area and pore size distribution (IUPAC Technical Report). Pure Appl. Chem..

[61] Tan Y., Sun Z., Meng H., Han Y., Wu J., Xu J., Xu Y., Zhang X. (2019). Efficient and selective removal of congo red by mesoporous amino-modified MIL-101(Cr) nanoadsorbents. Powder Technol..

[62] Far H.S., Hasanzadeh M., Najafi M., Rabbani M. (2022). Magnetic metal–organic framework (Fe_3_O_4_@MIL-101) functionalized with dendrimer: Highly efficient and selective adsorption removal of organic dyes. J. Inorg. Organomet. Polym. Mater..

[63] Chen Q., He Q., Lv M., Xu Y., Yang H., Liu X., Wei F. (2015). Selective adsorption of cationic dyes by UiO-66-NH_2_. Appl. Surf. Sci..

[64] Li L., Liu X.L., Geng H.Y., Hu B., Song G.W., Xu Z.S. (2013). A MOF/graphite oxide hybrid (MOF: HKUST-1) material for the adsorption of methylene blue from aqueous solution. J. Mater. Chem. A.

[65] Shao Y., Zhou L., Bao C., Ma J., Liu M., Wang F. (2016). Magnetic responsive metal–organic frameworks nanosphere with core–shell structure for highly efficient removal of methylene blue. Chem. Eng. J..

