# Supplementary material for: Template Method for Synthesizing Hierarchically Porous MIL-101(Cr) for Efficient Removal of Large Molecular Dye
Source: Materials (Basel). 2022 Aug 20;15(16):5763. doi: 10.3390/ma15165763 (PMC9416310; doi:10.3390/ma15165763)
Supplement: Supplementary file 1 [file materials-15-05763-s001.zip › materials-1856910-supplementary.pdf]

Template method for synthesizing hierarchically porous MIL-101(Cr) for efficient removal of large molecular dye

Minmin Zou, Hexin Zhu, Ming Dong, Tian Zhao*

School of Packaging and Materials Engineering, Hunan University of Technology, Zhuzhou 412007, China

Abstract: As one of the most important prototypical chromium-based MOFs, MIL-101(Cr) is well studied and widely employed in various scientific fields. However, due to its small capture window sizes and curved internal apertures, the application in large molecular removal is quite limited, and attributed to high stability and high synthetic temperature (> 200 ℃), it's very hard to achieve hierarchically porous MIL-101(Cr). In this paper, the hierarchically porous MIL-101(Cr) involving high macro-meso-/micro-pores ratio was designed and synthesized by using acetic acid as an additive and silicon dioxide (SiO_2) nanoparticles as a template. The optimal hierarchically porous MIL-101(Cr) (A-4) possessed high specific surface area (2693 $m^2 \cdot g^{-1}$) and abundant macro-meso-porous structure with the addition of SiO_2 of 200 mg. Compare with the control sample (A-0) with less macro-meso-porous structure, A-4 showed good adsorption properties for both Coomassie Brilliant Blue R-250 (CBB, 82.1 mg g^{-1}) and Methylene blue (MB, 34.3 mg g^{-1}) dyes, which was 1.36 times and 9.37 times higher than that of A-0. Moreover, A-4 also had good recyclability and the removal rate of CBB was still higher than 85% after five cycles of adsorption.

Keywords: MIL-101(Cr), SiO_2, dye adsorption, template

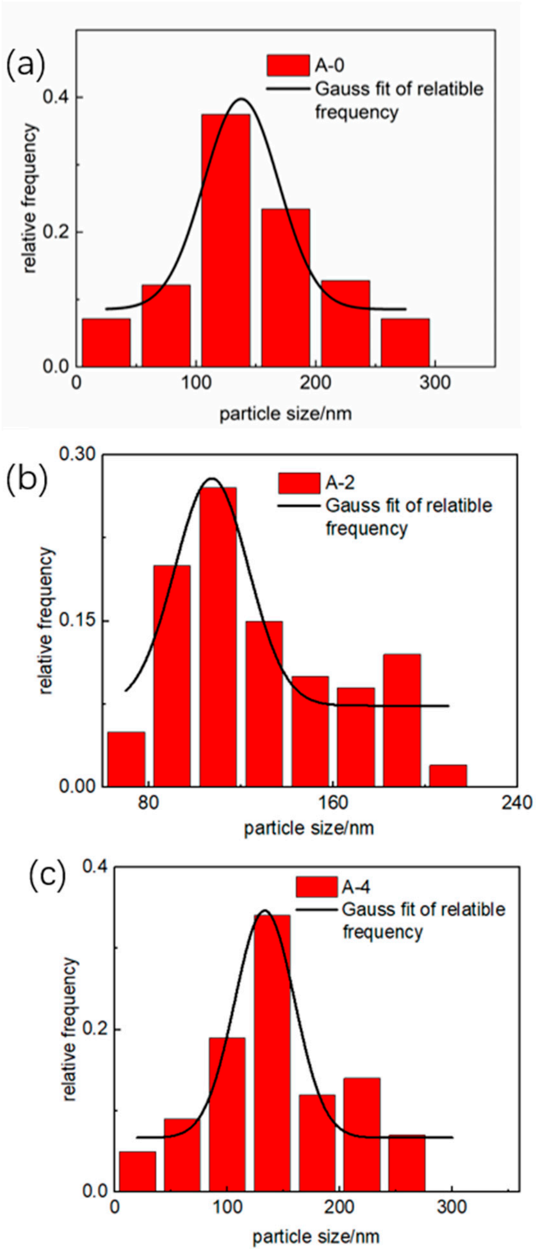

Figure S1. Particle size distribution of the samples: a) A-0, b) A-2, c) A-4.

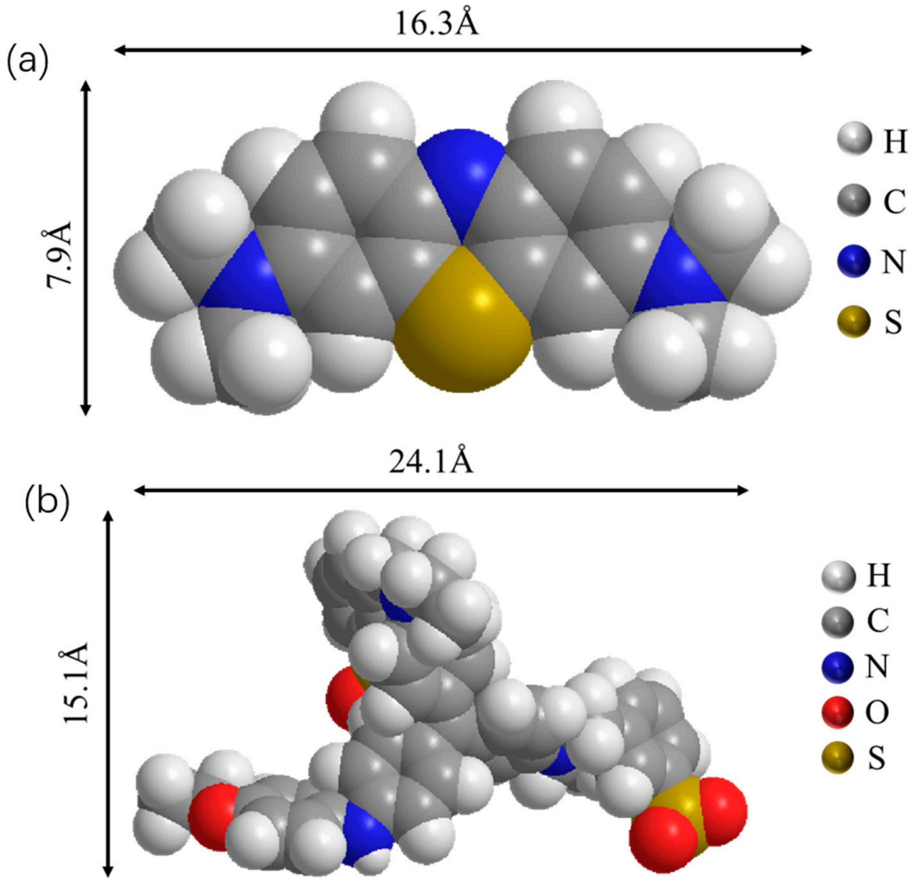

Figure S2. Molecular diagrams of a) methylene blue and b) coomassie brilliant blue dye.

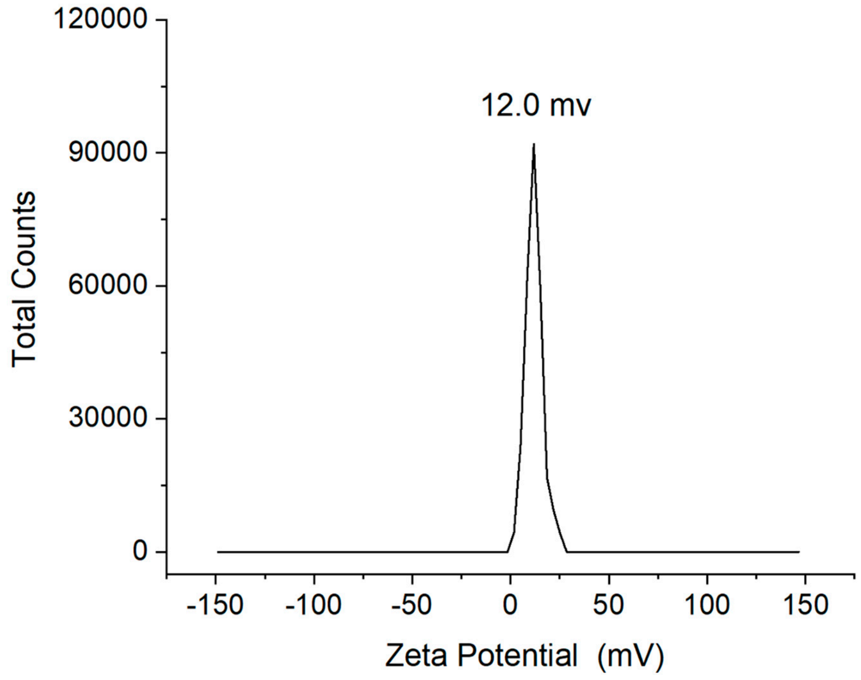

Figure S3. Zeta potential of A-4.

Table S1. Fitting parameters of Langmuir, Freundlich and Temkin models for sample A-4.

		Parameter	CBB	MB
Adsorption kinetics	Pseudo-first-order	$q_{e,Exp}$ (mg g^{-1})	158.3	54.33
		$q_{e,Cal}$ (mg g^{-1})	99.3	42.8
		k_1 (min^{-1})	0.0468	0.0402
		R^2	0.936	0.956
	Pseudo-second-order	$q_{e,Exp}$ (mg g^{-1})	158.3	54.33
		$q_{e,Cal}$ (mg g^{-1})	180.5	65.9
		k_2 (g mg^{-1} min^{-1})	4.5×10^{-4}	7.7×10^{-4}
		R^2	0.993	0.989
Adsorption isotherm	Freundlich	1/n	0.2493	0.4560
		k_F (mg g^{-1} (L $mg^{-1})^{1/n}$)	177.4	8.545
		R^2	0.689	0.888
	Langmuir	$q_{m,Exp}$ (mg g^{-1})	647.5	114.7
		$q_{m,Cal}$ (mg g^{-1})	666.7	146.2
		k_L (L mg^{-1})	0.110	0.011
		R^2	0.999	0.982

Table S2. Adsorption capacities (q_{max}) of MB on several adsorbents.

Adsorbent	Dyes	Uptake (mg g^{-1})	Ref.
MIL-101(Cr)	MB	4.24	[48]
HP-MIL-101(Cr)	MB	11.23	
NH_2-MIL-101(Cr)	MB	33	[61]
bulk phase MIL-101(Cr)	MB	12.9	[21]
nano-sized MIL-101(Cr)	MB	17.5	
MIL-101@PPI	MB	97.9	[62]
UiO-66-NH_2	MB	96.45	[63]
Fe_3O_4-COOH/HKUST-1	MB	118.6	[64]
Fe_3O_4@MIL-100(Fe)	MB	73.8	[65]
MIL-101(Cr)	MB	146.2	This work

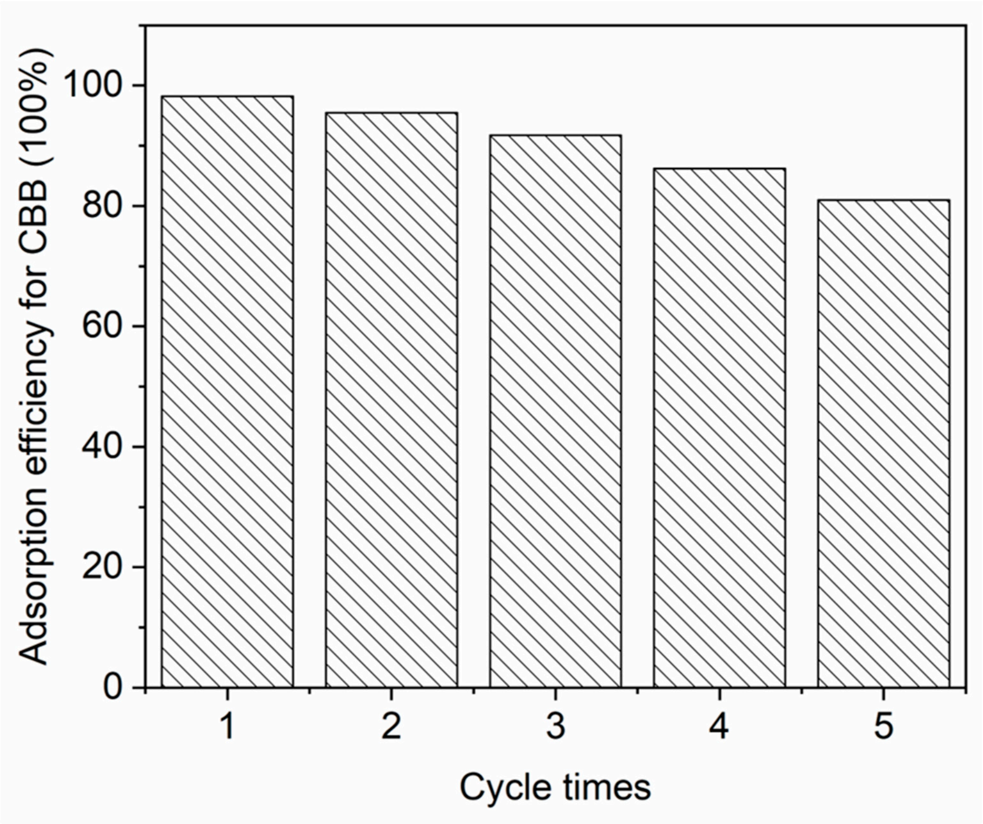

Figure S4. Cycle adsorption efficiency of A-4 adsorbed CBB.
